# A Reconstruction Method Based on AL0FGD for Compressed Sensing in Border Monitoring WSN System

**DOI:** 10.1371/journal.pone.0112932

**Published:** 2014-12-02

**Authors:** Yan Wang, Xi Wu, Wenzao Li, Yi Zhang, Zhi Li, Jiliu Zhou

**Affiliations:** 1 College of Electronics and Information Engineering, Sichuan University, Chengdu, Sichuan, China; 2 Department of Computer Science, Chengdu University of Information Technology, Chengdu, Sichuan, China; 3 College of Computer Science, Sichuan University, Chengdu, Sichuan, China; Xiamen University, China

## Abstract

In this paper, to monitor the border in real-time with high efficiency and accuracy, we applied the compressed sensing (CS) technology on the border monitoring wireless sensor network (WSN) system and proposed a reconstruction method based on approximately *l_0_* norm and fast gradient descent (AL0FGD) for CS. In the frontend of the system, the measurement matrix was used to sense the border information in a compressed manner, and then the proposed reconstruction method was applied to recover the border information at the monitoring terminal. To evaluate the performance of the proposed method, the helicopter sound signal was used as an example in the experimental simulation, and three other typical reconstruction algorithms 1)split Bregman algorithm, 2)iterative shrinkage algorithm, and 3)smoothed approximate *l_0_* norm (SL0), were employed for comparison. The experimental results showed that the proposed method has a better performance in recovering the helicopter sound signal in most cases, which could be used as a basis for further study of the border monitoring WSN system.

## Introduction

Border monitoring plays an important role in the national defense. The traditional border monitoring system consists of security checkpoints and border troops, which suffers from intensive human involvement. Moreover, as the long stretches of borders and the complexity of the terrain, the difficulty of manual patrolling is increased. To minimize the need for human support and monitor the border in real-time with high accuracy, multiple surveillance technologies, which complement each other, are required [1]. In recent years, the wireless sensor network (WSN) has been widely used in many military applications, which can help to monitor the border environmental information timely [2].

While the potential benefits of border monitoring system based on WSN are significant, several research challenges should be addressed before practical realization. Since the detection range of the borders is significantly wide, the border monitoring WSN system usually contains thousands of senor nodes, which would generate massive amount of data. Therefore, one major challenge is how to effectively collect and transmit the massive data [3]. Due to network bandwidth limitation and energy consumption of sensor nodes in WSN, the researchers usually wish to reconstruct the original signal from a small amount of data in practical application. Moreover, with the massive data obtained from thousands of sensor nodes, the conventional signal processing framework based on the Nyquist sampling theorem would bring great difficulties to storage and transmission. Compressed Sensing (CS) theory which has been used in many fields [4]-[8] provides a solution for the challenges.

CS comprises a collection of methods of representing a signal on the basis of a limited number of measurements and then recovering the signal from these measurements [9]. The problem of how to effectively recover the original signal from the compressed data plays a significant role in the CS framework. Currently, there exists several reconstruction algorithms which are defined either in the context of convex optimization, or greedy approaches. Basis pursuit (BP) is a popular mathematical optimization problem which is based on constrained *l_1_* norm minimization, and the split Bregman method is an effective technique for solving a variety of *l_1_*-regularized optimization problems [11]. Several reconstruction algorithms based on constrained *l_p_* norm minimization with *p*<1 have also been proposed [12], [13]. Furthermore, a signal reconstruction algorithm based on the optimization of a smoothed approximate *l_0_* norm (SL0) is studied in [14] where simulation results are compared with corresponding results obtained from several existing algorithms. The results favor the use of the approximate *l_0_* norm.

In this paper, we launch the application research on border monitoring based on CS theory. Gradient descent is extensively used in optimization problem, which is particularly suitable for inverse problems involving large-scale data sets [15]. Aiming at getting more information from fewer measurements and obtaining higher reconstruction accuracy, a novel signal reconstruction method based on approximate *l_0_* norm and fast gradient descent (AL0FGD) is proposed. The paper is organized as follows. In Section 2, we design the network architecture of border monitoring WSN system and introduce the theoretical basis of the proposed method. A simple sinusoidal signal is constructed as an experimental example to preliminarily study the validity of the proposed method in Section 3. In Section 4, we take an actual border monitoring sound signal (helicopter) as an example, and based on several experiments, the validity and superiority of the proposed method are demonstrated. Finally, Section 5 presents discussion and conclusion.

## Methodology

**Figure 1:**
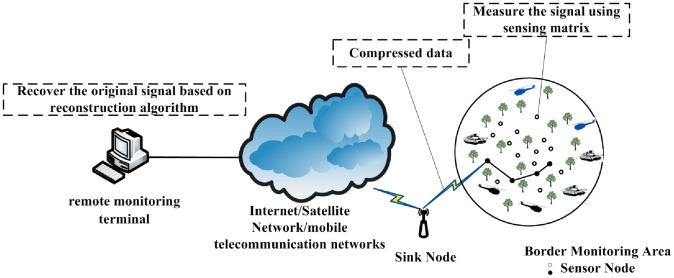
Network architecture diagram of the border monitoring WSN system.

It is known that WSN is a multihop and self-organized network formed from a large amount of wireless sensor nodes by wireless communication [16]. High energy efficiency is one of the most important requirements for WSN design. In this paper, CS theory is employed to reduce the energy consumption and prolong the lifecycle of the border monitoring WSN system. To be more exact, for the sensor nodes, the measurements matrix (Φ) is used to measure the border monitoring information in a compressed manner, which can vastly reduce the number of measurements that need to be stored and transmitted. Then the compressed and encoded data is sent to remote monitoring terminal via Internet, satellite network or mobile telecommunication networks. After that, the signal reconstruction algorithm is employed to recover the original border monitoring information at the base station where sufficient computing resources are available. Fig.1 shows the network architecture diagram of the border monitoring WSN system.

Since the proposed reconstruction method for CS is based on the approximate *l_0_* norm and fast gradient descent, in the following sections (2.1, 2.2 and 2.3), we will give the theoretical basis of the CS, the approximate *l_0_* norm, and the proposed method based on AL0FGD, respectively.

### 2.1 Compressed sensing

In the framework of compressed sensing, the signals probed are firstly assumed to be sparse or compressible in some basis. Consider a complex-valued signal ***x*** which itself may or may not be sparse in the canonical basis but is sparse or approximately sparse in an appropriate basis Ψ. That is,
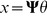
(2.11)where *θ* is sparse or approximately sparse. A central idea of the CS theory is about how a signal is acquired: the acquisition of signal ***x*** of length *n* is carried out by measuring *m* projections of ***x*** onto sensing vectors {φ*_i_^T^, i = 1, 2,…, m*}: *y_i_ = φ_i_^T^x* for *i* = *1, 2,…, m*. For sensing efficiency, we wish to collect a relatively much smaller number of measurements, that is, one requires that *m* be considerably smaller than *n* (*m<<n*), hence the name compressed sensing. This data acquisition mechanism is at the core of a CS system that marks a fundamental departure from the conventional data acquisition-compression-transmission-decompression framework: the conventional framework collects a vast amount of data for acquiring a high-resolution signal, then essentially discard most of the data collected (in the Ψ domain) in the compression stage, while in CS the data is measured in a compressed manner, and the much reduced amount of measurements are transmitted or stored economically, and every bit of the measurements are then utilized to recover the signal using reconstruction algorithms. The data acquisition process in CS framework is described by
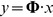
(2.12)


According to (2.11), (2.12) can be written as 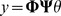 (the size of the sparsifying basis or sparse matrix Ψ is *n×n*). Typically with *m<n*, the inverse problem is ill-posed [17]. However, the sparest solution of (2.12) can be obtained by solving the constrained optimization problem
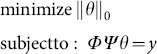
(2.13)where 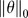 is the *l_0_* norm defined as 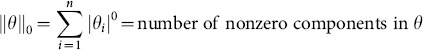



Unfortunately, it turns out that (2.13) is a problem of combinatorial complexity: finding solution of (2.13) requires enumerating subsets of the dictionary to identify the smallest subset that can represent signal ***x***, the complexity of such a subset search grows exponentially with the signal size *n* [18]. A key result in the CS theory is that if ***x*** is *r*-sparse, the waveforms in {φ*_i_^T^, i = 1, 2,…, m*} are independent and identically distributed random waveforms, and the number of measurements, *m*, satisfies the condition
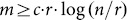
(2.14)where *c* is a small positive constant, then signal ***x*** can be reconstructed by solving the convex problem
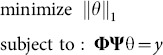
(2.15)where 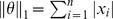 [9].

### 2.2 Approximate *l_0_* norm

Several greedy algorithms [19], [20] have been shown to enjoy exact reconstruction property, generally with less computational complexity. However, these algorithms require more measurements ({φ*_i_^T^, i = 1, 2,…, m*}) for exact reconstruction than the basis pursuit method, which turns out to be quite restrictive for many practical problems. Several researchers have recently studied new algorithms in the other direction. Specifically, the *l_1_* norm is replaced with the *l_p_* norm, where 0<*p*<1 [12], [13]. Then the problem of (2.15) become
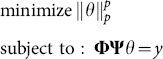
(2.21)where 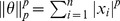. With *p*<1, the problem of (2.21) becomes nonconvex and multiple local solutions exist. However, if the problem is solved with sufficient care, we can obtain improved results related to those obtained by solving the problem in (2.15) [12]. In reference [14], the signal recovery problem is achieved by minimizing a smoothed approximate *l_0_* norm (SL0) of *θ* subject to 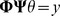, namely,
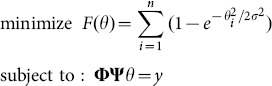
(2.22)where σ>0 is a parameter. This problem can be solved by using an algorithm based on the steepest-descent approach. It was found that this algorithm can offer improved signal reconstruction performance and computational complexity compared to several existing algorithms.

### 2.3 The proposed method based on AL0FGD

Because of the complexity of the terrain and environment, an important feature of the border monitoring target signal is the presence of contamination by some form of noise. Here note that for notion convenience we use ***x*** to denote the sparse signal vector that was denoted by *θ* in the noise-free counterpart of the above model. Signal ***x*** may be estimated from noisy measurement ***y*** by solving the minimization problem, as follows.
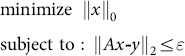
(2.31)where *A* is a sensing matrix of size *m* by *n* given by *A = ΦΨ*. According to (2.22), (2.31) is often found more natural to study the closely related problem [21]

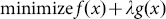
(2.32)where 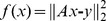 and 
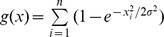
, and parameter *λ*>0 acts as a tradeoff between the sparsity of ***x*** and approximation error.

We begin by considering a simple problem of minimizing function *f(x)* which is smooth and convex. In the *k*th iteration of a conventional gradient method, known as the gradient descent method, iterate ***x*** *_k-1_* is update to 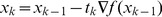
(2.33)where *t_k_*>0 is a scalar known as step size. According to Taylor expansion, *f(x)* can be written as 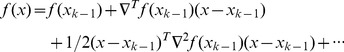
(2.34)


It can be easily verified that iterate ***x*** *_k-1_* specified by (2.33) may be interpreted as the solution of a simple quadratic problem:
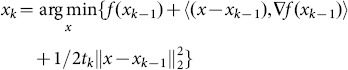
(2.35)


By neglecting constant terms, (2.35) can be written as
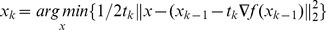
(2.36)


Therefore, the problem of compressed sensing of noisy signals formulated by (2.32) becomes
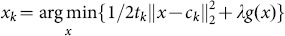
(2.37)where 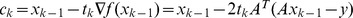.

Then, input 
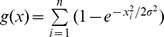
 into (2.37), we obtain 
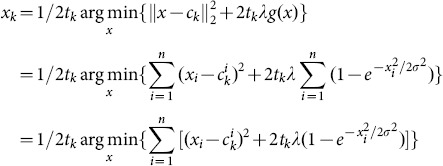
(2.38)


Because both the two addends in (2.38) are separable, i.e. each of them is mere the sum of *n* nonnegative terms and each of these terms involves only a single variable, the iterate ***x*** *_k_* in (2.37) can be computed exactly by minimizing each element of 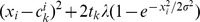
(assuming *c_k_* in (2.37) has been calculated).

For convenience, we let
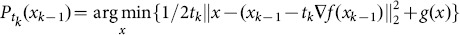
(2.39)


Then the primary step of the proposed method becomes
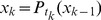
(2.40)


It can be shown that this method based on approximate *l_0_* norm and gradient descent (AL0GD) converges to a solution of (2.32) if the step size is selected to satisfy 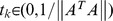. However, the convergence speed of AL0GD is shown to be slow. According to the fundamental principle of the fast gradient descent algorithm [22], we further improve AL0GD to obtain faster convergence speed. The final reconstruction method based on approximate *l_0_* norm and fast gradient descent (AL0FGD) is outlined below.

The proposed reconstruction method based on AL0FGD

Input: sensing matrix A, λ, *k*, σ

Step 0: Take 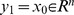, 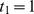


Step *k* (*k* ≥ 1) compute

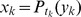
by solving the problem in (2.39)

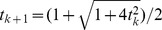

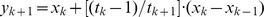

In the next section, we will use the experiment to comparatively investigate the convergence speeds of AL0GD and AL0FGD.

## An Experimental Example

**Figure 2:**
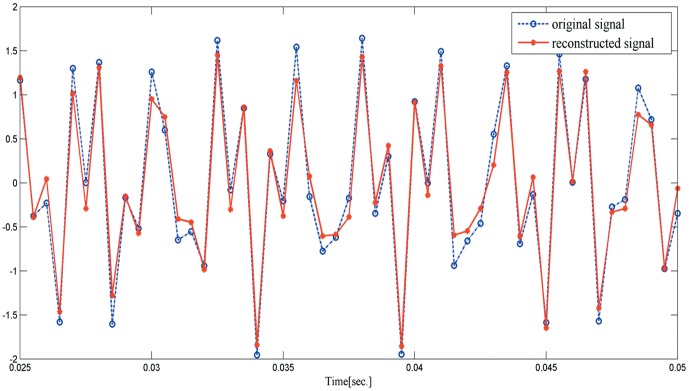
Comparison between the original and reconstructed signals using AL0FGD.

In order to preliminarily evaluate the validity of the proposed method, the signal in this section is constructed by sampling the analog signal
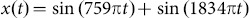
(3.1)at 2000Hz. In this experiment, a certain amount of white Gaussian noise is added to the original signal, and the standard deviation of white noise is set to 0.2. The number of measurements (*m*) is set to 340, and *t_k_* is fixed to 0.9. With *λ* = 0.35 and 30 iterations, we use the proposed reconstruction method to recover the signal. The reconstructed result over time interval [0.025, 0.05] is shown in Fig. 2 in comparison with the original signal ***x***. Here we mark the constructed signal ***x***∧.

**Figure 3:**
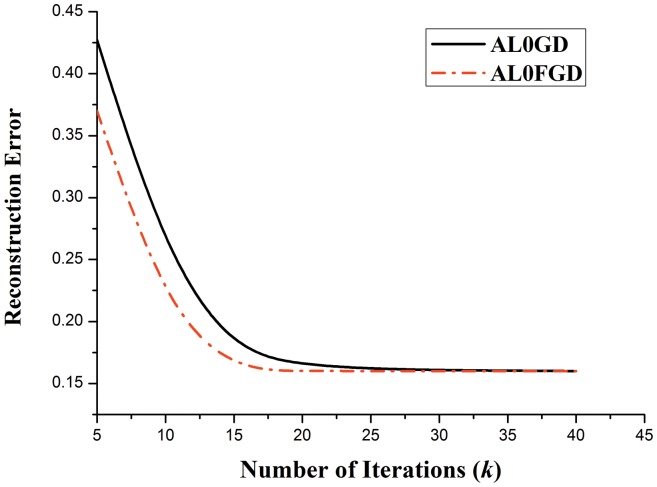
The convergence situation of AL0GD and AL0FGD with the increase of *k*.

As can be seen from Fig.2, the proposed method (AL0FGD) shows good performance in reconstructing the original signal. To study the convergence speeds of AL0GD and AL0FGD, the convergence situations of the two methods are compared. Fig. 3 shows the convergence situation of the two methods with the number of iterations increases, where the horizontal axis represents the number of iterations *k* while the vertical axis represents the reconstruction error *r* (
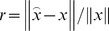
).

The comparison result of Fig. 3 shows that the convergence speed of AL0FGD is much faster than AL0GD, which is in accord with the theory in Section 2.3.

However, the signals in the border monitoring WSN system are much more complex than the simple sinusoidal example of (3.1). Whether the proposed reconstruction method can be applied to process the border monitoring signals needs to be further discussed. In the following section, the actual border monitoring signal will be studied to evaluate the validity and superiority of the proposed reconstruction method.

## Experimental Results

The actual border monitoring signal considered in this paper is the sound signal (e.g. helicopter sound). Currently, in most of the border monitoring systems, the video monitoring technology and the radar technology are used. However, these technologies have some disadvantages, such as high demand to the environment, the existence of blind areas and poor concealment. If the sound identification technology could be applied on this region, combined with video monitoring and radar technologies, the monitoring probability will be further elevated [23]–[25]. Therefore, amount of sound sensor nodes would be deployed in the border monitoring areas. Here we take the helicopter sound signal as an example to evaluate the performance of the proposed method in border monitoring WSN system.

**Figure 4:**
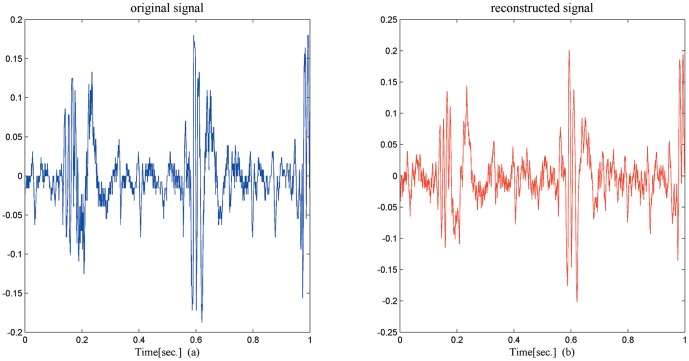
The original signal and reconstructed signal over time interval [0, 1].

**Figure 5:**
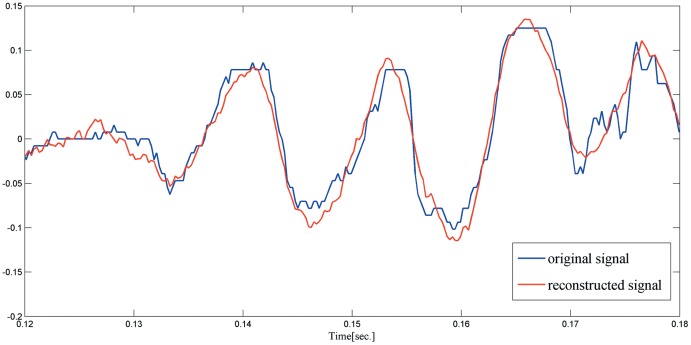
The original signal and reconstructed signal over time interval [0.12, 0.18].

One of the key notions in the CS theory is sparsity which expresses the idea that the “information rate” of a continuous-time signal may be much smaller than that suggested by its bandwidth, and it is found that many natural and man-made signals admit sparse representations when expressed in an appropriate basis Ψ [26]. In the experiment, the sparse matrix Ψ is an orthonormal basis constructed by1-D discrete Fourier transform, and the measurement matrix Φ is generated by orthonormalizing *n* vectors sampled independently and uniformly on the unit sphere. For the helicopter sound, the length of the signal is set to 4096, the input parameters *λ* and *k* are set to 0.35 and 40, respectively. Then the sparse matrix Ψ of size 4096 by 4096 is firstly applied to test the sparsity of the helicopter sound signal. The result shows that the number of the nonzero entries is only 352 with an appropriate threshold, indicating that the helicopter sound signal is sparse in the discrete Fourier domain. To preliminarily study the reconstruction performance of the proposed method, the number of measurements of Φ is set to 1200. Fig. 4 shows the comparison results between the original signal and reconstructed signal over time interval [0, 1]. In order to show the comparison more clearly, the results of the two signals is magnified over time interval [0.12, 0.18] (246 length) as shown in Fig. 5.

From the above experimental results, it seems that the proposed reconstruction method has convincing results in recovering the original helicopter sound signal in border monitoring WSN system.

In order to fully understand the proposed method, three aspects of works are further performed: 1) the performances for recovering the helicopter sound signal of AL0FGD and other CS reconstruction algorithms are comparatively studied, 2) since the complexity of the practical border monitoring areas, the sounds would contain numerous noises which cannot be ignored. Therefore, the performance of the proposed method in the noisy environment is further studied, 3) as the real-time performance is an important requirement of the border monitoring WSN system, the computational complexity plays a significant role of the reconstruction algorithm. Under the premise of the high reconstructive accuracy, whether the proposed method has a faster computation speed than other algorithms is a problem need to be further discussed.

### Comparative study on the performance of AL0FGD

**Figure 6:**
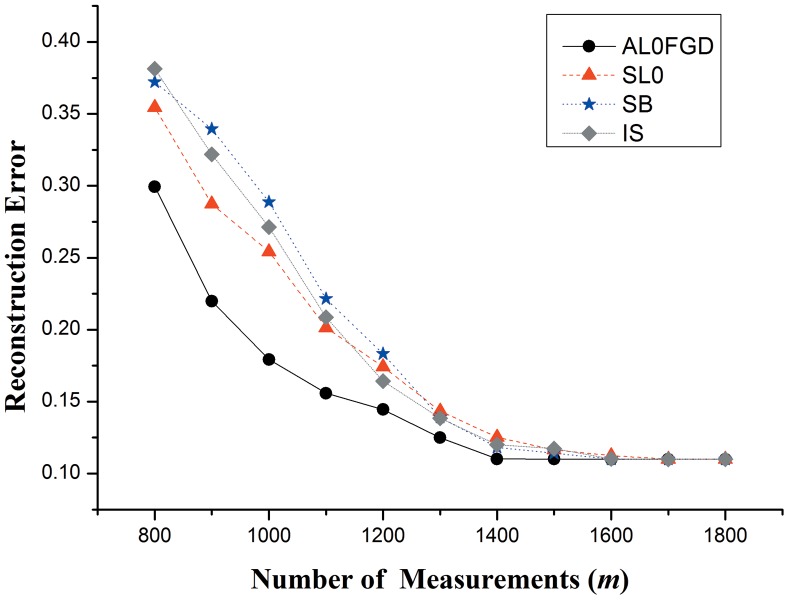
Comparison of construction error by the IS, SB, SL0 and AL0FGD algorithms over 50 runs.

To study the performance of the proposed method, we take some other reconstruction algorithms for comparison. As mentioned in Introduction, for BP optimization problem, split Bregman method is an effective technique for solving a variety of *l_1_*-regularized optimization problems. Moreover, iterative shrinkage algorithms have been proposed to handle a class of convex optimization problems arising in inverse problem [27]–[29]. Therefore, in the following experiments, three reconstruction algorithms: 1) split Bregman algorithm (SB) 2) iterative shrinkage algorithm (IS) 3) smoothed approximate *l_0_* norm (SL0) are employed for comparison. The number of measurements *m* is chosen from 800 to 1800. In order to make the comparison clear, the plots of reconstruction error (*r*) as a function of the number of measurements (*m*) is obtained as shown in Fig. 6.

Fig. 6 shows that the helicopter sound signal can be well recovered by all the four algorithms when the number of measurements reaches 1600. However, when the number of measurements is less than 1500, the superior reconstruction quality of the proposed method is quite evident. In the practical border monitoring WSN system, fewer measurements can increase the compression degree when sampling the signals, which can significantly improve the transmission efficiency and reduce the energy consumption of the system. Therefore, the experimental results show that the proposed method could have the highest transmission efficiency and lowest energy consumption of the system in practice, compared with IS, SB and SL0.

### Performance evaluation of AL0FGD in the noisy environment

The Gaussian white noise is added into the helicopter sound signals in the subsequent experiment. Here we set the number of the measurements to 1500. Table 1 shows the reconstruction error with the increase of signal-to-noise ratio (SNR) using four reconstruction algorithms.

**Table:** **Table 1.** Reconstruction error (*r*) of IS, SB, SL0 and AL0FGD with the increase of SNR.

Reconstruction algorithms	Reconstruction error (*r*) under different SNR						
	0 db	5 db	10 db	15 db	20 db	25 db	30 db
IS	0.5601	0.3679	0.2147	0.1601	0.1372	0.1290	0.1157
SB	0.4521	0.3326	0.2138	0.1582	0.1320	0.1254	0.1141
SL0	0.5213	0.3522	0.2231	0.1654	0.1382	0.1284	0.1165
AL0FGD	0.4120	0.2832	0.1954	0.1532	0.1315	0.1220	0.1112

As can be seen from Table 1, the reconstruction error decreases gradually with the increase of SNR. On the other hand, even though all four algorithms cannot perform well under the 0db SNR (the error is up to more than 0.4), it is still evident that, with the same SNR, the reconstruction error of the proposed method is lower than those of the other three algorithms.

### Computational complexity of AL0FGD

**Figure 7:**
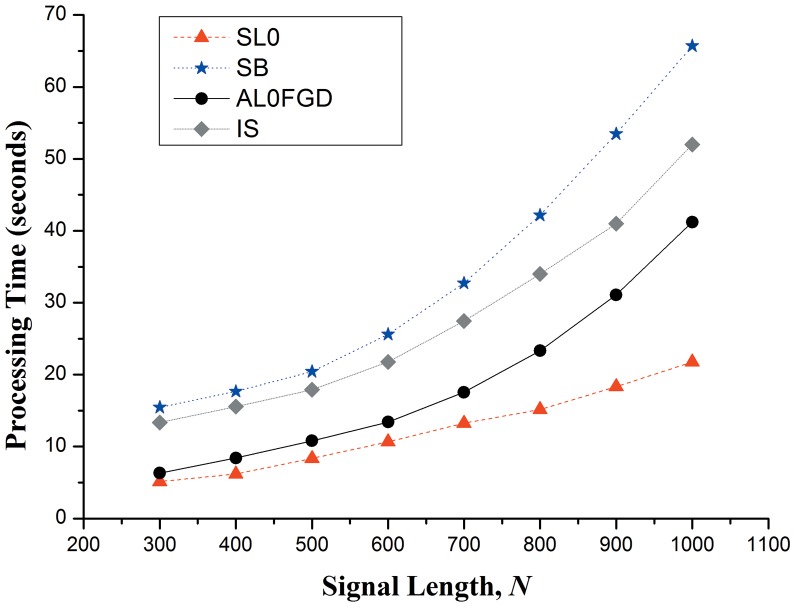
Average processing time required by the three algorithms over 50 runs.

The computational complexity of the algorithms in this paper is measured by the elapsed processing time. In the following experiment, we intercept the helicopter sound signals of different lengths (from 300 to 1000). The processing time is measured on a laptop with an Intel i3-2310M 2.1GHz processor using MATLAB commands *tic* and *toc*. Fig. 7 shows the average processing time required by the four reconstruction algorithms over 50 runs.

As can be seen from Fig. 7, the processing time of SL0 is the shortest among the four algorithms with the same signal length. Meanwhile, even though the convergence rate of AL0FGD is a little slower than that of SL0, it is obviously faster than those of SB and IS.

Synthetically considering the above experiments, it can be concluded that the performance of the proposed method is better than those of IS, SB and SL0 algorithms in most cases for reconstructing the helicopter sound information.

**Figure 8:**
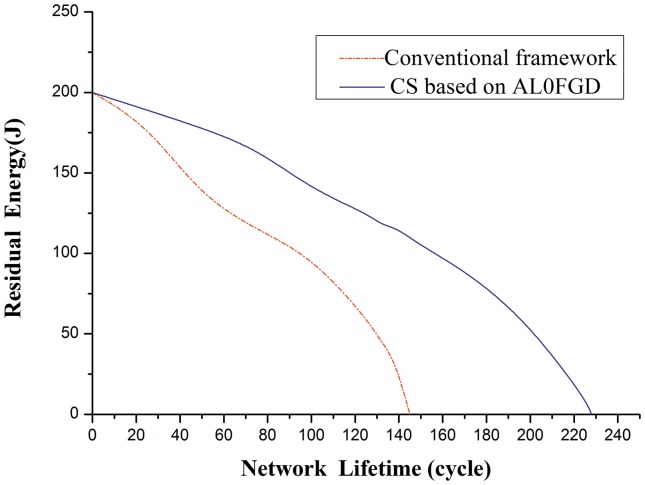
Comparison of the residual energy with the network lifetime.

However, as to the entire border monitoring WSN system, the sensor network lifetime mainly depends on the energy consumption due to the difficulty in charging batteries [30]. Next, in the same sensor network, we compare the energy performances of the network using CS theory based on AL0FGD and that using the conventional framework based on Nyquist sampling theorem. The initial energy of the network is set to 200 J. The comparison of the residual energy with the network lifetime is showed in Fig. 8.

Fig. 8 shows that the energy consumption ratio of the WSN system using conventional framework is much quicker than that using the CS theory based on AL0FGD, indicating that the border monitoring WSN system based on CS theory can save network energy and extend the life cycle of network more effectively. Moreover, as the amount of data to be transferred is greatly reduced based on the AL0FGD, the transmission speed would be significantly increased.

## Conclusion

The application of the CS theory on the border monitoring WSN system is a novel research field. In this paper, we assumed an ideal system which can accomplish the task of data acquisition, processing, and transmitting by the network. In the frontend of the network system, the measurement matrix of CS was used to measure the border monitoring information to obtain the compressed data. To recover the information more efficiently at the monitoring terminal, we proposed a novel reconstruction method based on AL0FGD for CS. Then we took the helicopter sound signal as an example to evaluate the performance of the proposed method, and the performance of AL0FGD was further compared with three other reconstruction algorithms (IS, SB and SL0). Comparison results showed that the proposed method can get more information from fewer measurements and perform better under the noisy environment. However, there are still some defects of the proposed method. For one thing, the reconstruction error was still high under strong noise and the computational efficiency of the proposed method was not as good as SL0. For another thing, we only considered the helicopter sound signal as an example to evaluate the performance of the proposed reconstruction method in this paper, however, the signal types of the border monitoring system are various, the performance of the proposed method for recovering other border monitoring sound signals or image information need to be further studied. Our future work will be focused on the optimization method of the algorithm and obtaining high reconstruction accuracy under strong noise, in order to meet the high requirement of the border monitoring WSN system. Moreover, more types of border monitoring signals will be studied to evaluate and improve the proposed method.

## Supporting Information

Helicopter S1 **The sound signal of helicopter 1.**
(WAV)

Helicopter S2 **The sound signal of helicopter 2.**
(WAV)

Helicopter S3 **The sound signal of helicopter 3.**
(WAV)

Helicopter S4 **The sound signal of helicopter 4.**
(WAV)

Helicopter S5 **The sound signal of helicopter 5.**
(WAV)
